# High levels of surgical antibiotic prophylaxis: Implications for hospital-based antibiotic stewardship in Sierra Leone

**DOI:** 10.1017/ash.2022.252

**Published:** 2022-07-07

**Authors:** Sulaiman Lakoh, Joseph Sam Kanu, Sarah K. Conteh, James B.W. Russell, Stephen Sevalie, Christine Ellen Elleanor Williams, Umu Barrie, Aminata Kadie Kabia, Fatmata Conteh, Mohamed Boie Jalloh, Gibrilla F. Deen, Mustapha S. Kabba, Aiah Lebbie, Ibrahim Franklyn Kamara, Bobson Derrick Fofanah, Anna Maruta, Christiana Kallon, Foday Sahr, Mohamed Samai, Olukemi Adekanmbi, Le Yi, Xuejun Guo, Rugiatu Z. Kamara, Darlinda F. Jiba, Joseph Chukwudi Okeibunor, George A. Yendewa, Emmanuel Firima

**Affiliations:** 1 College of Medicine and Allied Health Sciences, University of Sierra Leone, Freetown, Sierra Leone; 2 Ministry of Health and Sanitation, Government of Sierra Leone, Freetown, Sierra Leone; 3 Sustainable Health Systems, Freetown, Sierra Leone; 4 Infectious Disease Research Network, Freetown, Sierra Leone; 5 34 Military Hospital, Freetown, Sierra Leone; 6 World Health Organization Country Office, Freetown, Sierra Leone; 7 Department of Medicine, College of Medicine, University of Ibadan, Nigeria; 8 Department of Medicine, University College Hospital, Ibadan, Nigeria; 9 Tropical Disease Prevention and Control Center, Freeown, Sierra Leone; 10 US Center for Disease Control and Prevention, Freetown, Sierra Leone; 11 World Health Organization Regional Office for Africa, Brazzaville, Congo; 12 Department of Medicine, Case Western Reserve University School of Medicine, Cleveland, Ohio, United States; 13 Division of Infectious Diseases and HIV Medicine, University Hospitals Cleveland Medical Center, Cleveland, Ohio, United States; 14 Johns Hopkins Bloomberg School of Public Health, Baltimore, Maryland, United States; 15 Clinical Research Unit, Department of Medicine, Swiss Tropical and Public Health Institute, Basel, Switzerland; 16 University of Basel, Basel, Switzerland; 17 SolidarMed, Maseru West, Lesotho; 18 Centre for Multidisciplinary Research and Innovation, Abuja, Nigeria

## Abstract

**Objective:**

Despite the impact of inappropriate prescribing on antibiotic resistance, data on surgical antibiotic prophylaxis in sub-Saharan Africa are limited. In this study, we evaluated antibiotic use and consumption in surgical prophylaxis in 4 hospitals located in 2 geographic regions of Sierra Leone.

**Methods:**

We used a prospective cohort design to collect data from surgical patients aged 18 years or older between February and October 2021. Data were analyzed using Stata version 16 software.

**Results:**

Of the 753 surgical patients, 439 (58.3%) were females, and 723 (96%) had received at least 1 dose of antibiotics. Only 410 (54.4%) patients had indications for surgical antibiotic prophylaxis consistent with local guidelines. Factors associated with preoperative antibiotic prophylaxis were the type of surgery, wound class, and consistency of surgical antibiotic prophylaxis with local guidelines. Postoperatively, type of surgery, wound class, and consistency of antibiotic use with local guidelines were important factors associated with antibiotic use. Of the 2,482 doses administered, 1,410 (56.8%) were given postoperatively. Preoperative and intraoperative antibiotic use was reported in 645 (26%) and 427 (17.2%) cases, respectively. The most commonly used antibiotic was ceftriaxone 949 (38.2%) with a consumption of 41.6 defined daily doses (DDD) per 100 bed days. Overall, antibiotic consumption was 117.9 DDD per 100 bed days. The Access antibiotics had 72.7 DDD per 100 bed days (61.7%).

**Conclusions:**

We report a high rate of antibiotic consumption for surgical prophylaxis, most of which was not based on local guidelines. To address this growing threat, urgent action is needed to reduce irrational antibiotic prescribing for surgical prophylaxis.

## Highlights

- For the first time, we have used the World Health Organization (WHO)–defined daily dose (DDD) to estimate antibiotic consumption in surgical prophylaxis in 4 hospitals located in 2 geographic regions of Sierra Leone.
- There were a high rates of antibiotic use (723, 96%) and consumption (117.9 DDD per 100 bed days) in surgical antibiotic prophylaxis, which is consistent with local guidelines in only 410 (54.3%) surgeries.
- The most commonly used antibiotic was ceftriaxone 949 (38.2%) with a consumption of 41.6 DDD per 100 bed days.
- The Access antibiotics accounted for 72.7 DDD per 100 bed-days (61.7%), whereas consumption of the Watch antibiotics was 45.2 DDD per 100 bed-days (38.3%). No Reserve antibiotics were utilized.
- Factors associated with preoperative antibiotic prophylaxis were the type of surgery, wound class, and consistency of surgical antibiotic prophylaxis with local guidelines. Postoperatively, surgical wound type and consistency of surgical antibiotic prophylaxis with local guidelines were important factors associated with antibiotic use.

***Introduction***

The discovery of antibiotics heralded a major success in the treatment and prevention of infectious diseases.^1^ However, the positive impact of antibiotics on health and other development sectors is threatened by the global increase in the levels of antimicrobial resistance (AMR) and the decline in the discovery of new antibiotics.^2,3^

AMR is a growing public health problem because its global burden continues to increase and could reach an unprecedented level of 10 million deaths per year by 2050 if global efforts to control it are not mobilized.^4^ In 2019 alone, the global deaths attributable to AMR were 1.27 million, making AMR the leading infectious cause of death after coronavirus disease 2019 (COVID-19) and tuberculosis (TB).^5^

Antibiotic consumption has increased in low- and middle-income countries (LMICs) due to improving economies and high burden of infectious diseases.^4^ Consequently, the burden of AMR in LMICs is much higher than in high-income countries.^4^ This fact is reflected in the recent revelation that the West African subregion has the highest mortality burden due to bacterial AMR.^5^

AMR prolongs hospital stays, increases morbidity and mortality, diverts financial resources that could be used to improve health, and threatens efforts to combat infectious diseases.^6,7^ In response to the numerous health and safety threats posed by AMR to populations, recent global efforts have been mobilized to prevent an increase in the burden of AMR.^8^ Despite these efforts, many gaps in the global AMR prevention and control remain, including the lack of routine surveillance data on antibiotic use and consumption in many countries in sub-Saharan Africa.^2^ At the national level, efforts are underway to reduce the AMR burden in Sierra Leone with support from the Fleming Fund.^9^ Even so, our previous studies have shown many challenges in the prevention and control of AMR in the country. Some of these challenges include a high burden of extended-spectrum β-lactase (ESBL)–producing gram-negative bacteria and gaps in hand hygiene implementation.^10–13^

The abuse and overuse of antibiotics, including surgical antibiotic prophylaxis, is one of the main reasons for the increased burden of AMR.^14,15^ The use of antibiotics is essential to preventing surgicalsite infections. In many cases in low-income countries, however, the use of antibiotics for surgical prophylaxis is often inappropriate, unnecessary, and can exacerbate antibiotic resistance.^16,17^ Therefore, data on the use and consumption of antibiotics for surgical prophylaxis in medical institutions are crucial for formulating and implementing policies to address the problem of inappropriate use of antibiotics for surgical prophylaxis.^2^ Using this information, hospital-based antimicrobial stewardship programs are able to minimize or prevent the growing threat of AMR and can address the problem of limited access to essential antibiotics that are needed to treat infectious diseases affecting humans.^7^

In this study, we assessed antibiotic use and consumption in surgical prophylaxis in 4 hospitals located in 2 geographic regions of Sierra Leone to inform antimicrobial stewardship interventions and monitor antibiotic consumption trends. The specific study objectives were (1) to determine the prevalence of antibiotic use for surgical prophylaxis, (2) to define the predictors of antibiotic use for surgical prophylaxis, (3) to evaluate antibiotics consumption using the 2021 Anatomical Therapeutic Chemical (ATC) and defined daily dose (DDD) method,^18,19^ and (4) to identify patterns of antibiotic consumption using the WHO Access, Watch, and Reserve (AWaRe) classification system.^18,19^

## Methods and materials

### Study design

In this study, we used a prospective cohort design to assess antibiotic use and consumption for surgical prophylaxis.

### Study setting

We selected 4 hospitals for the study because they share similar characteristics with many secondary- and tertiary-care health facilities in Sierra Leone: Connaught Hospital (CH), Miliatary Hospital (MH), Makeni Government Hospital (MGH), and Lumley Government Hospital (LGH). LGH (42 beds) is a secondary-care facility, but the remaining 3 hospitals provide tertiary care. CH (300 beds), MH (181 beds), and LGH are in Freetown, the capital of Sierra Leone, with a population of 1 million, whereas MGH (207 beds) is located in a regional city, ∼170 km from the capital, with a catchment population of 606,544 or ∼8.6% of the Sierra Leonean population.^20^ Although MGH is the only government-owned hospital in its catchment area, Freetown has 8 hospitals that provide surgical services, with a total capacity of 890 beds. The study was conducted on 523 hospital beds (69%) in Freetown.

With the exception of the national referral hospital (CH), which provides surgical services to the nonpregnant population, the remaining 3 hospitals provide surgical services to both pregnant and nonpregnant populations. All 4 hospitals are owned by the government of Sierra Leone. On average, each week, 20 adult patients undergo surgery at CH, 17 at MGH, 10 at MH, and 4 at LGH.

### Study population and study duration

The study included all adult (18 years or older) patients who received elective and emergency surgical services at 4 hospitals between March 2021 and October 2021 as part of a surgical-site infection surveillance program. Because the surveillance program excluded patients with amputation or other bone surgery, data on prophylactic surgical antibiotics in these procedures could not be included in the study.

### WHO standardized classification systems for antibiotic use and consumption

Various standardized classification systems were employed to categorize antibiotic use or consumption patterns. The WHO AWaRe framework classifies antibiotics used for surgical prophylaxis as listed in the WHO Model List of Essential Medicine.^18,21^ The Access antibiotics have a low resistance potential and should always be available to treat common bacterial infections.^18^ Because the Watch antibiotics are the highest-priority antimicrobials, they are key targets for monitoring the progress of hospital-based antimicrobial stewardship programs.^18^ The Reserve antibiotics are the last-line, used to treat multidrug-resistant infections and are the target of national AMS programs.^18,21^ When assessing antibiotic use in surgical prophylaxis, data were disaggregated by indication for surgical prophylaxis and related to the class of wound.

Antibiotic substances were further classified using the 2021 ATC classification system.^21^ Using the ATC system, antibiotics used for surgical prophylaxis were classified according to their anatomical, therapeutic and/or pharmacological, and chemical properties into all levels of classification at the subgroup level (ie, level 3, ATC3) and the chemical substance level (ie, level 5, ATC5).^21^ Antibiotic consumption was evaluated using the DDD per 100 bed days for each of the 4 hospitals.^21^ In assessing antibiotic consumption, aggregated surgical antibiotic prophylaxis data were estimated for each hospital.

### Data collection and analysis

Trained nurses collected the data in the Epicollect software platform (Epic, Verona WI) from the patient files and patient and/or ward nurse interviews. After collection, the data were extracted into Microsoft Excel (Microsoft, Redmond, WA), cleaned, coded, and then transferred into Stata version 16 software (StataCorp, College Station, TX) for analysis. Descriptive statistics such as frequencies and percentages were used to summarize demographic and clinical characteristics of study participants, as well as antibiotic consumption. The proportion of indications and the type or dose of surgical antibiotic prophylaxis consistent with the local antibiotic guidelines were determined using descriptive statistics. Binary logistic regression was employed to identify factors associated with antibiotic use. Results were adjusted for hospital variables and are presented for the pre- intra-, and postoperative periods. Firth correction was applied where observations were zero. A *P* value <.05 was considered statistically significant.

The DDD values were calculated by converting the total amount of antibiotic dispensed into grams, which were then divided by the WHO-assigned DDD based on the 2022 version of the ATC/DDD index.^21^ To calculate DDD per 100 bed days, we divided consumption in DDD by bed days and multiplied by 100. A bed day was defined as an overnight stay in the hospital. We calculated the total DDD per 100 bed days in the sample and then determined the contributions to this total for the pre-, intra-, and postoperative periods, as well as for each antibiotic. We also calculated antibiotic consumption per 100 bed days for each hospital.

### Ethical approval

Ethical approval was obtained from the Sierra Leone Ethics and Scientific Review Committee of the Ministry of Health and Sanitation, Government of Sierra Leone. Written informed consent was obtained from each participants.

## Resuls

### Demographic characteristics of study participants

In total, 753 patients underwent surgery during the study period. Most had their surgeries at Connaught Hospital (n = 245, 32.5%); most patients were female (439, 58.3%); and the median patient age was 30 years (IQR, 10–87). The sociodemographic characteristics of patients in the 4 hospitals are summarized in Table 1.

**Table 1.** Demographic Characteristics of Study Participants

Parameter	Total, No. (%)	Connaught Hospital, No. (%)	Lumley Government Hospital, No. (%)	Military Hospital, No. (%)	Makeni Government Hospital, No. (%)
Overall total	753 (100)	245 (32.5)	93 (12.34)	182 (24.2)	233 (30.9)
**Sex**					
Female	439 (58.3)	74 (30.2)	72(77.4)	68(37.4)	225(96.6)
Male	314 (41.7)	171 (69.8)	21 (22.6)	114 (62.6)	8 (3.4)
**Age group**					
<25 y	211 (28.0)	36 (14.7)	29 (31.2)	44 (24.2)	102 (43.8)
25–44 y	392 (52.1)	121 (49.4)	53(57.0)	91(50.0)	127 (54.5)
45–64 y	113 (15.0)	67 (27.3)	10 (10.7)	34 (18.7)	2 (0.9)
≥65 y	37 (4.9)	21 (8.6)	1(1.1)	13(7.1)	2(0.9)
Median y (range)	30 (10–87)	38 (18–87)	28 (10–81)	34 (10–80)	25 (18–75)
**Marital status**					
Single	239 (31.7)	79 (32.2)	28 (30.1)	68 (37.4)	64 (27.5)
Married	489 (64.9)	143 (58.4)	65 (69.9)	112 (61.5)	169 (72.5)
Separated/divorced/widowed	25 (3.3)	23(9.4)	…	2 (1.1)	…
**Education**					
None	157 (20.8)	71 (29.0)	16 (17.2)	38 (20.9)	32 (13.7)
Primary	96 (12.8)	28 (11.4)	7 (7.5)	29 (15.9)	32 (13.7)
Secondary	370 (49.1)	106 (43.3	41 (44.1)	83 (45.6)	140 (60.1)
Tertiary	130 (17.3)	40 (16.3)	29 (31.2)	32 (17.6)	29 (12.5)
**Occupation**					
None	218 (29.0)	27 (11.0)	16 (17.2)	41 (22.8)	134 (57.5)
Student	116 (15.5)	30 (12.2)	21 (22.6)	53 (29.4)	12 (5.2)
Informal sector	303 (40.4)	143 (58.4)	44 (47.3)	43 (23.9)	73 (31.3)
Formal sector	98 (13.1)	37 (15.1)	11 (11.8)	36 (20.0)	14 (6.0)
Retired	16 (2.1)	8 (3.3)	1 (1.1)	7 (3.9)	…
**Alcohol use**					
No	676 (89.9)	209 (85.3)	85 (92.4)	149 (81.9)	233 (100)
Yes	76 (10.1)	36 (14.7)	7 (7.6)	33 (18.1)	…
**Cigarette smoker**					
No	688 (91.5)	206 (84.1)	86 (93.5)	163 (89.6)	233 (100)
Yes	64 (8.5)	39 (15.9)	6 (6.5)	19 (10.4)	…
**Other substances**					
No	747 (99.2)	239 (97.5)	93 (100)	182 (100)	233 (100)
Yes	6 (0.8)	6 (2.5)	…	…	…

### Clinical characteristics of study participants

The surgical procedures considered in this study were abdominal surgeries (n = 388, 51.5%), caesarean sections (n = 309, 41%), and nonabdominal surgeries (n = 56, 7.4%). The wound classes were clean (n = 584, 77.6%), clean contaminated (n = 138, 18.3%), and contaminated (n = 31, 4.1%). Although >90% of surgical wounds in LGH, MH, and MGH were clean, fewer than half of the surgical wounds at CH were clean (n = 115, 46.9%).

### Antibiotic use for surgical prophylaxis

Of the 753 total surgeries, 723 (96%) had at least 1 dose of antibiotics in at least 1 period (ie, pre-, intra-, or post-operative). Only 30 patients (4%) had surgery without any surgical antibiotic prophylaxis. In the preoperative period, 490 patients (65%) received antibiotics. Nearly all patients in MH and MGH (97.8% and 95.3%, respectively) received antibiotics during the preoperative period. Also, one-third (248, 33%) received antibiotics during surgery (ie, intraoperative period). In the postoperative period, 692 patients (92%) received antibiotics. Although CH administered antibiotics to 77% of its patients, LGH placed all of its patients on antibiotics, and almost all patients received antibiotics at MH and MGH (97.8% and 99.6%, respectively).

As shown in Table 2, only 410 patients (54.4%) had indications for surgical antibiotic prophylaxis consistent with local guidelines. Procedures with the most consistent indications for surgical antibiotic prophylaxis were reported in MGH (n = 211, 90.6%), making it the hospital with the highest compliance. Only 70 surgeries (28.6%) at CH had indications for antibiotic prophylaxis consistent with local guidelines.

**Table 2.** Clinical Characteristics of Study Participants

Parameter	Total, No. (%)	Connaught Hospital, No. (%)	Lumley Government Hospital, No. (%)	Military Hospital, No. (%)	Makeni Government Hospital, No. (%)
Overall total	753 (100)	245 (32.5)	93 (12.34)	182 (24.2)	233 (30.9)
**Type of surgery**					
Nonabdominal	56 (7.4)	45 (18.4)	3 (3.2)	7 (3.8)	1 (0.4)
Abdominal	388 (51.5)	200 (81.6)	30 (32.3)	133 (73.1)	25 (10.7)
Obstetric	309 (41.0)	…	60 (64.5)	42 (23.1)	207 (88.8)
**Presence of comorbidity**					
No	635 (84.3)	181 (73.9)	68 (73.1)	158 (86.8)	228 (97.8)
Yes	118 (15.7)	64 (26.1)	25 (26.9)	24 (13.2)	5 (2.2)
**Wound class**					
Clean	584 (77.6)	101 (41.2)	90 (96.8)	181 (99.4)	212 (91.0)
Clean contaminated	138 (18.3)	115 (46.9)	1 (1.1)	1 (0.6)	21 (9.0)
Contaminated	31 (4.1)	29 (11.8)	2 (2.1)	…	
**Preoperative antibiotics**					
No	263 (34.9)	194 (79.2)	54 (58.1)	4 (2.2)	11 (4.7)
Yes	490 (65.1)	51 (20.8)	39 (41.9)	178 (97.8)	222 (95.3)
**Intraoperative antibiotics**					
No	504 (67.0)	104 (42.5)	…	170 (93.4)	230 (98.7)
Yes	248 (33.0)	141 (57.5)	92 (100)	12 (6.6)	3 (1.3)
**Postoperative antibiotics**					
No	60 (8.0)	55 (22.5)	…	4 (2.2)	1 (0.4)
Yes	692 (92.0)	189 (77.5)	93 (100)	178 (97.8)	232 (99.6)
**Consistency of indication for antibiotic prophylaxis to the local guideline?**					
No	343 (45.6)	175 (71.4)	23 (24.7)	123 (67.6)	22 (9.4)
Yes	410 (54.4)	70 (28.6)	70 (75.3)	59 (32.4)	211 (90.6)
**Consistency of the type and dose of antibiotic used to the local guidelines?**					
No	309 (74.8)	33 (50.8)	25 (33.8)	37 (61.7)	214 (100)
Yes	104 (25.2)	32 (49.2)	49 (66.2)	23 (38.3)	…
Total bed days	5,042	2,185	471	1245	1,141

### Factors associated with antibiotics use

Using binary logistic regression analysis and adjusting for hospitals, the significant factors associated with preoperative surgical antibiotic prophylaxis were the type of surgery, wound class, and consistency of surgical antibiotic prophylaxis with local guidelines. Abdominal surgeries were nearly 3 times more likely to receive antibiotics for surgical prophylaxis, (aOR, 2.7; 95% CI, 1.1–6.7; *P* = .034) than nonabdominal surgeries. Compared to clean wounds, antibiotics were 6 times more likely to be used in clean-contaminated wounds (aOR, 6.3; 95% CI, 2.3–16.8; *P* < .001) and 47 times more likely in contaminated wounds (aOR, 47.4; 95% CI, 14.5–155.4; *P* < .001). Preoperative patients were >3-fold more likely to receive antibiotics when they are indicated in the local guidelines (aOR, 3.4; 95% CI, 2.1–5.7; *P* < .001]. As in the preoperative period, abdominal surgeries accounted for a greater likelihood of receiving antibiotics (aOR, 3.6; 95% CI, 1.9–7.2; *P* < .001) postoperatively than nonabdominal surgeries.

### Consumption of antibiotics

Of the 2,482 doses of antibiotics administered, 1,410 (56.8%) were given postoperatively. Preoperative and intraoperative antibiotic use was reported in 645 cases (26%) and 427 cases (17.2%), respectively. Among the hospitals, MH had the highest rate of antibiotic use (924 cases, 37.2%) followed by CH (635 cases, 25.6%), LGH (464 cases, 18.7%), and MGH (459 cases, 18.5%).

The most used antibiotic was ceftriaxone (949 cases, 38.2%) with consumption of 41.6 DDD per 100 bed days. Cefuroxime and clarithromycin were each administered only once (Table 4).

**Table 3.** Hospital-Adjusted Logistic Regression Showing Antibiotics Use in the pre- intra- and post- operative periods

Parameter	Preoperative Period				Intraoperative Period				Postoperative Period			
	Antibiotics Administered No (%) Yes (%)		aOR (95% CI)	*P* Value	Antibiotics Administered No (%) Yes (%)		aOR (95% CI)	*P* Value	Antibiotics Administered No (%) Yes (%)		aOR (95% CI)	*P* Value
**Sex**												
Female	112 (42.6)	327 (66.7)	1		316 (62.7)	122 (49.2)			22 (36.7)	416 (60.1)		
Male	151 (57.4)	163 (33.3)	0.9 (0.6–1.6)	.889	188 (37.3)	126 (50.8)	0.9 (0.6–1.5)	.792	38 (63.3)	276 (39.9)	1.3 (0.7–2.5)	.327
**Age group**												
<25 y	52 (19.8)	159 (32.5)	1		157 (31.1)	54 (21.8)			8 (13.3)	203 (29.3)		
25–44 y	127 (48.3)	265 (54.1)	1.2 (0.7–2.1)	.457	258 (51.2)	133 (53.6)	1.1 (0.6–2.1)	.781	30 (50)	362 (52.3)	0.8 (0.3–1.9)	.611
45–64 y	66 (25.1)	47 (9.6)	0.6 (0.3–1.3)	.212	66 (13.1)	47 (18.9)	0.8 (0.4–1.7)	.551	19 (31.7)	93 (13.4)	0.6 (0.2–1.6)	.317
≥65 y	18 (6.8)	19 (3.9)	0.9 (0.3–2.7)	.847	23 (4.6)	14 (5.7)	1.0 (0.4–2.6)	.987	3 (5.0)	34 (4.9)	1.5 (0.3–6.3)	.582
**Alcohol**												
No	227 (86.6)	449 (91.6)	1		455 (90.3)	220 (89.1)			51 (85)	624 (90.3)		
Yes	35 (13.4)	41 (8.4)	0.8 (0.4–1.7)	.519	49 (9.7)	27 (10.9)	0.7 (0.4–1.4)	.340	9 (15)	67 (9.7)	1.0 (0.4–2.2)	.948
**Presence of comorbidity**												
No	200 (76.0)	435 (88.8)	1		452 (89.7)	182 (73.4)			46 (76.7)	588 (85.0)		
Yes	63 (24.0)	55 (11.2)	1.3 (0.7–2.1)	.411	52 (10.3)	66 (26.6)	1.2 (0.7–2.1)	.483	14 (23.3)	104 (15.0)	1.1 (0.6–2.2)	.741
**Type of surgery**												
Nonabdominal	43 (16.4)	13 (2.6)	1		28 (5.6)	28 (11.3)			30 (33.3)	35 (5.1)		
Intra-abdominal	171 (65.0)	217 (44.3)	2.7 (1.1–6.7)	.034	231 (45.8)	156 (62.9)	1.2 (0.6–2.2)	.639	39 (65.0)	349 (50.4)	3.6 (1.9–7.2)	<.001
Obstetric	49 (18.6)	260 (53.1)	1.5 (0.5–4.7)	.483	245 (48.6)	64 (25.8)	0.6 (0.1–2.8)	.489	1 (1.7)	308 (44.5)	11.8 (0.7–205.5)	.089
**Wound class**												
Clean	165 (62.7)	419 (85.5)	1		439 (87.1)	144 (58.1)			42 (70)	541 (78.2)		
Clean-contaminated	89 (33.8)	49 (10.0)	6.3 (2.3–16.8)	<.001	60 (11.9)	78 (31.4)	2.9 (1.7–5.0)	<.001	18 (30)	120 (17.3)	3.1 (1.6–5.9)	<.001
Contaminated	9 (3.4)	22 (4.5)	47.4 (14.5–155.4)	<.001	5 (1.0)	26 (10.5)	7.1 (2.5–20.0)	<.001	0 (0)	31 (4.5)	34.9 (2.1–587.8)	.014
**Consistency of indication to guidelines**												
No	174 (66.2)	169 (34.5)	1	…	228 (45.2)	114 (46.0)			53 (88.3)	289 (41.8)		
Yes	89 (33.8)	321 (65.5)	3.4 (2.1–5.7)	<.001	276 (54.8)	134 (54.0)	3.4 (1.9–5.9)	<.001	7 (11.7)	403 (58.2)	4.3 (1.8–10.1)	.001

Note. OR, odds ratio; CI, confidence interval.

**Table 4.** Antibiotics Administered and ATC Code, AWaRe Category, Frequency of Administration, and Contribution to Total Antibiotic Consumption (DDD per 100 Bed Days)

Variable	ATC Code	AWaRe Category	Drug Administration (N=2,482), No. (%)	DDD Per 100 Bed Days (N=117.9), No. (%)
Amoxicillin	J01CA04	Access	17 (0.7)	1.1 (0.9)
Amoxicillin-clavulanate	J01CR02	Access	95 (3.8)	14.5 (12.3)
Ampicillin	J01CA01	Access	400 (16.1)	15.0 (12.7)
Ampicillin-cloxacillin	J01CR50	Access	165 (6.7)	9.5 (8.1)
Azithromycin	J01DB01	Watch	3 (0.1)	0.1 (0.08)
Ceftriaxone	J01DD04	Watch	949 (38.2)	41.6 (35.3)
Cefuroxime	J01DC02	Watch	1 (0.04)	0.1 (0.08)
Ciprofloxacin	J01MA02	Watch	10 (0.4)	1.0 (0.8)
Clarithromycin	J01FA09	Watch	1 (0.04)	0.1 (0.08)
Gentamycin	J01GB03	Access	64 (2.6)	2.2 (1.9)
Levofloxacin	J01MA12	Watch	12 (0.5)	1.9 (1.6)
Metronidazole	P01AB01	Access	765 (30.8)	30.8 (26.1)

Note. DDD, defined daily dose.

**Fig. 1.**
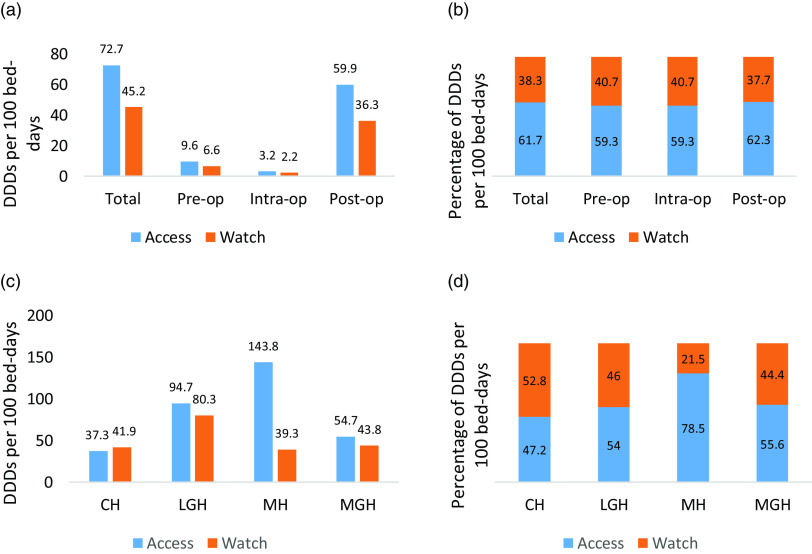
(a) Total antibiotic consumption presented as DDD per 100 bed days by WHO AWaRe category showing contributions from preoperative, intraoperative, and postoperative periods. (b) Proportion of total antibiotic DDD per 100 bed days by WHO AWaRe category during preoperative, intraoperative, and postoperative periods. (c) Antibiotic consumption and (d) percentage consumption by WHO AWaRe category in 4 hospitals in Freetown, Sierra Leone. Note. CH, Connaught Hospital; LGH, Lumley Government Hospital; MH, Military Hospital; and MGH, Makeni Government Hospital.

The overall antibiotic consumption was 117.9 DDD per 100 bed days. The Access antibiotics accounted for 72.7 DDD per 100 bed days (61.7%), whereas consumption for the Watch antibiotics was 45.2 DDD per 100 bed days (38.3%) (Fig. 1a and b). Of the overall antibiotic consumption in the study, clean wounds accounted for 70.8% (83.5 DDD per 100 bed days); clean-contaminated wounds accounted for 17.6% (20.8 DDD per 100 bed days); and contaminated wounds accounted for 11.5% (13.6 DDD per 100 bed days). Consumption of antibiotics was highest in MH at 183.1 DDD per 100 bed days and was least in CH at 79.2 DDD per 100 bed days (Fig. 1c).

The Access antibiotics had consistently higher total DDD per 100 bed days in the 3 periods (pre-, intra-, and postoperative). It was highest in the postoperative period at 59.9 DDD per 100 bed days. Its proportion ranged from 59.3% to 62.3% of total DDD per 100 bed days within the operative periods. Consumption rates for antibiotics in the Access category ranged from 47.2% in CH to 78.5% in MH. No antibiotics in the Reserve group were utilized. Other details on antibiotics consumption are shown in Figure 1.

## Discussion

To the best of our knowledge, this is the first large-scale surgical antibiotic prophylaxis study in Sierra Leone. In this study, most patients received antibiotics for surgical prophylaxis, higher than the levels reported in Ghana, Gambia, and Turkey.^22–24^ All 4 hospitals had a combined DDD of 117.9 per 100 bed days, which means that 1.179 DDD of antibiotic prophylaxis were administered for every procedure in these hospitals each day. The DDD reported in our study was higher than the DDD reported for surgical antibiotic prophylaxis in Nigeria (n = 17) and Indonesia (n = 30).^25,26^ The high rates of antibiotic use in our setting may be due to a lack of oversight of rational antibiotic prescribing. This rate may be reduced by a functional antibiotic stewardship system. In 2019, a national antibiotic consumption study in Sierra Leone reported a DDD of 19 per 1,000 inhabitants per day.^27^ Although a direct comparison with this study is somewhat inappropriate due to differences in methodology and study setting, national antibiotic consumption of 19 DDD was much lower than the 117.9 DDD reported in our study. The large differences between national and hospital-based data suggest that estimates of antibiotic consumption can provide better information if studies are performed at different levels.

We identified gaps in our estimate of antibiotic consumption, which if not addressed urgently, could hinder the effort of the country in meeting the UN Sustainable Development Goals (ie, targets 3.1–3.3 and 3.8, and goal 6), especially for reducing AMR.^28^ One of the priority gaps identified in this study is the high rate of surgical antibiotics prophylaxis that does not follow local guidelines. In nearly half of the patients, indications for antibiotic prophylaxis were inconsistent with the local antibiotic guidelines. Similarly, the dose or type of antibiotics used for surgical prophylaxis was inconsistent with local guidelines in 75% of the surgeries. This finding may be explained by the fact that although all the hospitals in this study have drugs and therapeutics committees, the committees have broader responsibilities, including procurement and supply chain management, and they cannot effectively carry out AMS activities. In 2017, the first antimicrobial guideline in Sierra Leone was developed at CH. Although there were plans to scale-up the use of these guidelines to other hospitals, their implementation has not been supported by local AMS activities, such as dedicated leadership and a multidisciplinary approach, which has hindered their effective use. Therefore, hospital administrators and policy makers should take urgent action to develop robust strategies to address the inappropriate surgical antibiotics prophylaxis in Sierra Leone. Functional hospital-based AMS programs are a good platform to protect available antibiotics and prevent the development of resistance.^29^ Nonetheless, establishing an AMS program in a low-income country like Sierra Leone requires practical action and sustainable funding. Hence, support from the government and its partners is needed to set up and sustain AMS activities in hospitals.

Our study highlights significant differences in the pattern of antibiotic consumption in surgical prophylaxis, which is expected given the varying levels of service across these 4 hospitals. Paradoxically, however, the DDD of the national referral hospital that admitted more sick patients was lower than that of the other hospitals. This finding may be due to differences in human resources required for the rational use of antibiotics for surgical prophylaxis. A national oversight system for rational antibiotic prescribing in hospitals can address this variability in prescribing patterns.

The most common antibiotics used for surgical prophylaxis in this study were ceftriaxone, metronidazole, and ampicillin. This finding is consistent with our previous study^30^ and with a study conducted in Nigeria,^25^ but it differs from a study conducted in Turkey^31^ and the WHO guidelines on surgical antibiotic prophylaxis.^32^ This fact adds to the background rationale that surgical antibiotic prophylaxis should follow local normative guidance.^33^

Antibiotics in the Access category are widely available and affordable and are used as first- or second-line treatment.^18^ Most antibiotics used for surgical prophylaxis in this study were in the Access group and none were in the Reserve group, probably due to the unavailability of drugs in this group or perhaps due to their high cost. However, this finding shows good prescribing practice because the WHO guidelines recommend that at least 60% of total antibiotic consumption in any particular setting should be in the Access group.^32^ In making this assertion, it is important to note that the AWaRe classification of antibiotics provides an overall national target and is not specifically designed to classify antibiotic use patterns in individual hospitals.^34^ Owing to their higher resistance potential, Watch antibiotics should be a key target for AMS interventions, especially in hospitals where ceftriaxone (cf, in the Watch group) is the most common antibiotic used for surgical prophylaxis.^35^

Our study had both strengths and limitations. A large sample size of patients who received surgical services from 4 different hospitals in 2 geographic regions of Sierra Leone was included in this study. Although not nationally representative, this evidence provides insight into the wider use of antibiotics for surgical prophylaxis and can be used to promote and implement AMS programs. We used local unpublished antibiotic guidelines developed in 2017 to determine the appropriateness of indications, doses, and types of antibiotics for surgical prophylaxis. Because these guidelines were not developed based on local evidence, it may not represent the true picture of surgical antibiotic prophylaxis in this setting. Also, the study population was largely unbalanced, which may have affected the representation of pregnant women. We did not assess surgical antibiotic prophylaxis in private hospitals, which can be done in future research.

In conclusion, we report a high rate of antibiotic consumption for surgical prophylaxis, most of which was not based on antibiotic guidelines. To address this growing threat to global health security, key stakeholders in the AMR and infection prevention and control response should take immediate steps to reduce irrational antibiotic prescribing for surgical prophylaxis by establishing local normative guidelines and training healthcare workers to follow the surgical antibiotic prophylaxis guidelines.
